# Prevalence and Factors Associated with Acute Kidney Injury in Sub-Saharan African Adults: A Review of the Current Literature

**DOI:** 10.1155/2022/5621665

**Published:** 2022-03-15

**Authors:** Charles Kangitsi Kahindo, Olivier Mukuku, Stanis Okitotsho Wembonyama, Zacharie Kibendelwa Tsongo

**Affiliations:** ^1^Faculty of Medicine, University of Goma, Goma, Democratic Republic of the Congo; ^2^Clinique Internationnale de Medecine Avancee au Kivu, Goma, Democratic Republic of the Congo; ^3^Institut Supérieur des Techniques Médicales de Lubumbashi, Lubumbashi, Democratic Republic of the Congo; ^4^Faculty of Medicine, University of Lubumbashi, Lubumbashi, Democratic Republic of the Congo; ^5^Faculty of Medicine, University of Kisangani, Kisangani, Democratic Republic of the Congo

## Abstract

Acute kidney injury (AKI) is a complex condition that can occur in both community and hospital settings and has many aetiologies. These aetiologies may be infectious, toxic, surgical, or related to the different management methods. Although it is a major public health problem worldwide, it must be emphasised that both its incidence and mortality rate appear to be very high in sub-Saharan African (SSA) countries compared to developed countries. The profile of AKI is very different from that of more developed countries. There are no reliable statistics on the incidence of AKI in SSA. Infections (malaria, HIV, diarrhoeal, and other diseases), nephrotoxins, and obstetric and surgical complications are the main aetiologies in Africa. The management of AKI is costly and associated with high rates of prolonged hospitalisation and in-hospital mortality.

## 1. Introduction

Acute kidney injury (AKI) refers to any sudden decrease in kidney function, which may be reversible if detected early enough [1, 2]. AKI is responsible for significant morbidity and mortality worldwide, but data on the global burden of disease are clearly lacking. AKI is a major problem frequently encountered in hospital patients and its incidence is increasing. This is due to the ageing population, but also to the wider use of therapeutic methods and/or diagnostic techniques with a risk of renal damage.

The occurrence of AKI is accompanied by an increase in the length of hospital stay and short- and long-term mortality [3, 4]. In sub-Saharan Africa (SSA), AKI is a complex problem in terms of demographics, financial, and medical resources. There are no reliable statistics on the incidence of AKI in SSA [5–8]. AKI can be community-acquired, resulting from poisoning, envenomation or infection prior to hospital admission, or hospital-acquired, occurring following hospital management [9–11]. Community-acquired AKI tends to occur in low-income countries and in young people with few comorbidities, whereas hospital-acquired AKI tends to occur in high-income countries and in older people (45–80 years) often with multiple comorbidities [12]. The proportion of AKI in SSA is unknown, but mortality is likely to be high due to poor access to health care [7, 13, 14]. In this literature review, we review recently published data on the demographics, causes, diagnoses, management, and prognosis of AKI in SSA.

## 2. Definition and Classification

The definition of AKI is most often based on the known imperfect creatinine level. AKI is classically defined as a sudden and persistent decrease in renal function [15]. AKI reflects the severity of clinical condition by the installation of uremic syndrome and a constellation of biological abnormalities whose intensity directly defines a therapeutic emergency. From a pathophysiological point of view, this phase is always preceded by renal aggressions (mainly haemodynamic and inflammatory), which if repeated can lead to irreversible renal tissue damage and, in the final stage, to dysfunction. In the past, there was noconsensus on the best method of assessing renal function and no consensus on the exact thresholds for the diagnosis of AKI. This complex problem not only leads to conflicting publications, but is also considered a major obstacle to research in this area. This led to the absence of reliable estimates of the incidence of AKI in SSA [16, 17].

Before 2004, there were more than 30 definitions of AKI with no real consensus, making comparison of the different studies difficult or impossible. A consensual definition was therefore necessary to unify practices. Several definitions and classifications were therefore proposed [18, 19]. They are all based on the idea that minor changes in renal function lead to significant consequences on the outcome of patients in the short term and probably in the long term. This new term includes the whole spectrum of acute kidney failure from minor changes in kidney function to the need for extra renal replacement therapy [15]. With this in mind, the definitions of AKI have been reviewed and standardised, taking into account these early insult phenomena and allowing for harmonisation of practices and better characterisation of the degree of renal failure.

### 2.1. Risk, Injury, Failure, Loss, End-Stage (RIFLE)

The Acute Dialysis Quality Initiative (ADQI) expert group proposed in 2004 a definition of AKI based on the creatinine level and urine output. This is the RIFLE classification, which stands for “risk of renal dysfunction,” “injury of the Kidney,” “failure of kidney function,” “lossbof kidney function,” and “end-stage of kidney disease.” This classification has been proposed to define AKI but also to classify their severity. The first 3 stages correspond to 3 levels of AKI of increasing severity: Risk of renal damage (R), renal damage (I), renal function impairment (F) [20, 21]. The last two stages represent two levels of clinical evolution describing persistent renal failure requiring extra-renal purification for more than four weeks (L) and chronic or permanent renal failure requiring extra-renal purification for more than three months (E). This RIFLE classification has the advantage of determining the number of patients still requiring renal replacement therapy at four weeks and three months, and the creatinine standards according to ethnicity and gender [19, 21–24].

### 2.2. Acute Kidney Injury Network (AKIN)

The AKIN classification, published in 2007, is a modified version of the RIFLE scale. It has only three stages and is based exclusively on changes in creatinine and diuresis. In this classification, the Risk, Injury and Failure stages have been replaced by AKIN stages 1, 2, and 3 respectively. A creatinine elevation of at least 26 mol/l has been added to the definition of stage 1 and patients who require renal replacement therapy is now classified as stage 3. The loss and end-stage renal disease classes have been removed [20, 21].

### 2.3. Kidney Disease: Improving Global Outcomes (KDIGO)

The AKI definitions proposed during the 2012 KDIGO guidelines were for adult and paediatric patients (Table 1). However, they currently seem difficult to apply to children. Indeed, the RIFLE and AKIN criteria on which the KDIGO guidelines are based do not take into account the size of the patients and are based on plasma creatinine values, which poses a problem in children whose muscle mass is much lower than in adults. For this reason, a paediatric version of RIFLE (pRIFLE) based on estimated plasma creatinine clearance and diuresis were proposed by Akcan-Arikan et al. [25]. In these modified criteria, AKI is defined by the existence of at least one of the following criteria: >25% decrease in estimated plasma creatinine clearance, diuresis <0.5 ml/h for 8 hours [16, 17, 20]. In this classification, estimated creatinine clearance is calculated using the paediatric Schwartz formula [24] and compared to a reference value of 100 ml/min/1.73 m^2^ if no previous value is known for the patient, which is often the case in paediatrics. This pRIFLE classification was then validated prospectively in various studies involving paediatric intensive care patients or immediate postoperative patients, particularly in cardiac surgery. By extension, the severity of AKI is also based on the pRIFLE [26, 27]. The KDIGO classification represents an optimised synthesis of the two pre-existing combined classifications (RIFLE and AKIN), developed by international expert groups of nephrologists to characterise the severity of AKI. A large body of literature since the publication of the RIFLE and AKIN classifications shows that these classifications are indeed well correlated with the severity of AKI since the morbidity (risk of progression to the need for renal replacement therapy, length of stay in the intensive care unit/hospital, risk of progression to chronic kidney injury) and mortality increase proportionally with the stage of these classifications. Once the diagnosis of AKI has been made, it is necessary to determine the severity of the AKI in order to determine its prognosis [20, 21, 27].

## 3. Incidence and Prevalence of AKI in Sub-Saharan Africa

The burden of AKI in SSA is unknown, but mortality is likely to be high due to poor access to health care [5, 8]. Epidemiological data on AKI are available from a few countries or hospitals and remain very diverse in SSA where it is difficult to interpret data on the incidence of AKI due to the non-uniformity of cohorts (some studies include only adults, others mix adults and children), the difference in definitions used in the diagnosis of AKI and the different methods of reporting results. As a result, there are no reliable statistics on the incidence of AKI in Africa [5]. The estimate of AKI frequencies in SSA countries is based on data from single-centre studies in urban settings whose findings do not allow for generalization. However, it should be noted that these studies make important contributions to understanding the burden of AKI and draw attention to this disorder [7].

In this literature review, we focused on the epidemiology of AKI in adult inpatients. Many previous studies have attempted to provide estimates of the incidence of AKI, but it is important to note that there is significant variability between studies due to the type of study, the definition of AKI, the location of patient recruitment and the length of the observation period for AKI occurrence [10]. Despite the use of new classifications, the epidemiology of AKI remains difficult to define. It varies greatly according to the population studied, depending on whether we are assessing a general population, a population of hospitalised patients or a population of patients requiring care in an intensive care unit; moreover, within the intensive care units themselves, it will depend on the type of pathologies managed locally (cardiac surgery, septic shock, major burns, etc.) [28].

Although data are still fragmentary, considerable efforts have recently been made to collect epidemiological data on AKI in adult patients in SSA. Osman et al. found that AKI accounted for 5.7% of all adult admissions to a tertiary teaching hospital in Sudan over a one-year period [29]. A South African study by Fenna et al. recorded a 6.2% prevalence of AKI in 18,781 adult patients admitted to a tertiary hospital in Cape Town [30]. In Benin, Vigan et al. showed that AKI was present in 11.8% of all adult admissions in two large hospitals in Cotonou [31]. In a prospective cohort of 253 Zimbabwean adults newly admitted to a large tertiary hospital in Harare, Gilbert et al. recorded 14.2% AKI [32]. In Malawi, Evans et al. in a prospective study of general medical admissions to a tertiary hospital in Blantyre found 17.2% of patients with AKI [33]. A prospective study including 2402 internal medicine and intensive care admissions in a tertiary referral hospital in Cameroon reported an overall incidence of AKI of 22.3% and an annual incidence of 15 per 100 patient-years [34].

Recent African studies that have focused on describing the incidence of AKI in adult intensive care settings have reported incidences exceeding 50% of admissions: 52.7% in seven intensive care units in Kinshasa (in the Democratic Republic of the Congo [DRC]) [35], 52.9% in the intensive care unit of a tertiary teaching hospital in Zambia [36], 55.3% in the intensive care unit of a tertiary hospital in northern Tanzania [37], and 58.5% in the intensive care unit of a tertiary hospital in Port Elizabeth, South Africa [38]. These reported frequencies of AKI in hospitalised and intensive care patients in Africa appear to be much higher than those found in published reports on AKI in developed countries [6].

We believe that the extent of AKI in SSA may be underestimated due to environmental and socioeconomic conditions that contribute significantly to the occurrence of AKI. The population of SSA faces several etiological factors of AKI such as various intoxications (traditional treatments, chemicals,…), envenomations (exposure to snake bites, spider bites and insect bites), infectious diseases (dengue, malaria, Ebola virus disease,…) and obstetric complications [7]. To these can be added the inability to measure renal function in patients at risk of AKI due to low financial resources but also the underequipment of health facilities. All these factors contribute to the underestimation of the extent of this condition.

## 4. Factors Associated with AKI in Sub-Saharan Africa

The main factors associated with AKI in SSA vary between regions, but most of these factors appear to be found in a large number of countries. A wide range of patient- and context-specific factors are found in the occurrence of AKI in African adults. AKI in SSA is particularly multifactorial and often caused by environmental and socioeconomic conditions.

### 4.1. Demographic Factors

#### 4.1.1. Age

The age of onset of AKI in developed countries is mainly advanced and increasing; this is in contrast to SSA countries where the population is young with a single etiology and where socioeconomic level and age are closely related to the risk of developing AKI [30, 39, 40]. But it is important to note that hospital-acquired AKI tends to occur in the elderly, often with multiple comorbidities (diabetes mellitus, hypertension, and cardiovascular disease) [34].

#### 4.1.2. Sex

Male sex appears to be a risk factor for AKI in most African studies [8, 32, 38]. The fact that male patients predominate in African series is often attributed by some authors to poor access of females to health care. In contrast, others suggest that there is a biological or environmental sensitivity to AKI in males [8]. It should also be emphasised that, as shown by two recent studies from southern Africa, this is not a case of female underrepresentation but of a highly significant association between male sex and the occurrence of AKI [32, 38].

#### 4.1.3. Race

Several studies had found that black subjects face a higher risk of AKI than other races [41–43]. After adjustment for potential confounders (sociodemographic factors, cardiovascular risk factors, etc.), the risk of AKI in black subjects still remains high. Demirjian points out that key mediators such as diabetes, high blood pressure and obesity, all of which are more prevalent in blacks, only partially account for the risk attributed to race [42].

### 4.2. Preexisting Conditions (Comorbidities)

As in developed countries, the major risk factors for AKI in SSA are comorbidities such as diabetes mellitus, hypertension, cardiovascular disease, and obesity, which are a corollary of the progressive westernisation of SSA society [32–34, 44–46]. As Olowu et al. point out, the rapid urbanisation of SSA combined with an increase in the prevalence of these non-infectious comorbidities have significantly influenced the clinical features of AKI [8].

Apart from the above comorbidities, Chronic Kidney Disease (CKD) has also been shown to be an independent risk factor for the development of AKI. Various African studies have shown that CKD is one of the strongest predictors of AKI [32, 38, 47]. After logistic regression, a Zimbabwean study found that the odds of developing AKI were 3.3 for elevated blood pressure and 6.0 for CKD [32]. In a Sudanese cohort, cardiovascular disease (adjusted odds ratio (AOR) = 3.4; 95% Confidence Intervals (95% CI): 1.2–9.4), diabetes mellitus (AOR = 2.6; 95% CI: 1.2–6.0) and hypertension (AOR = 2.4; 95% CI: 1.2–5.4) were significant predictors of the development of AKI [44].

Another comorbidity, and not the least, that is found in sub-Saharan Africans is Sickle Cell Disease (SCD) [48]. AKI occurs in 4–10% of patients hospitalised with SCD [49, 50]. Several renal disturbances have been described during SCD [47, 51–54]. During SCD, cyclical episodes of renal ischaemia and ischaemia-reperfusion occur, predisposing to recurrent subclinical and clinical AKI [54]. In a cohort of 30 Malian SCD patients, Fongoro et al. recorded 40% of AKI [55]. In Burkina Faso, Traoré et al. reported 6.3% renal damage in a series of 79 adults with SCD [56].

### 4.3. Infections

The various literature reviews conducted show that the main aetiologies of AKI in SSA are dominated by infections [5, 8, 34]. Several specific infections play an important role in the development of AKI. Where data are available, sepsis has been shown to be the main cause of AKI on presentation to hospital in Africa; far fewer cases develop AKI after hospital admission [6]. Due to the rapid urbanisation of SSA, millions of people live in communities with poor sanitation or overcrowded and unsanitary living conditions that favour the persistence or re-emergence of various infectious diseases (bacterial, viral and parasitic), thus providing a chronic infection environment conducive to the development of AKI. Tropical infections are an important cause of renal dysfunction, and some diseases have a high incidence of AKI [57]. Sepsis is a major cause of AKI in SSA and occurs in 26% to 50% of cases [34, 48, 58–60]. The main causes of sepsis are cholera, other diarrhoeal diseases, typhoid and malaria. After malaria (especially *Plasmodium falciparum*), dengue fever is the most frequent infectious cause of AKI, along with leptospirosis and acute bacterial gastroenteritis [5, 61]. These tropical diseases can be prevented by effective personal and environmental hygiene measures. Thus, vector control is of paramount importance in the prevention of dengue virus transmission. Such control would contribute to the reduction of the incidence of AKI associated with these infections. Some of the infections causing AKI have been frequently and recently reported in adults in SSA.

#### 4.3.1. Malaria

The number of malaria cases was estimated to be 241 million in 2020, and almost 95% of malaria cases were recorded in Africa. Malaria is caused by a protozoan of the genus Plasmodium. There are four species that are pathogenic to humans: P. falciparum, P.malariae, P. vivax and P. ovale [47]. AKI in malaria is most common in infections caused by Plasmodium falciparum, and manifestations include oliguria, severe metabolic acidosis, hypercatabolic state, and fluid and electrolyte disturbances, such as hyponatremia and hyperkalaemia [48, 57, 62]. The prevalence of AKI in P. falciparum infection ranges from 1% to 4% in endemic areas. However, it can reach 60% in severe infections [47, 63]. In a study of 104 Tanzanians with malaria, Muhamedhussein et al. showed that the prevalence of AKI was 26% and 18.3% respectively at 48 hours and 7 days of admission [64]. In an Angolan series of 86 patients hospitalised for malaria, Sacomboio et al. reported 42% AKI [65]. Histopathological findings in malaria-associated AKI includeacute tubular necrosis, interstitial nephritis, inflammatory interstitial infiltrate, oedema and glomerulonephritis, and the pathogenesis is associated with blockage of the renal microcirculation, haemodynamic factors and hypovolaemia [57, 66, 67]. AKI may be associated with massive parasitemia, intravascular haemolysis, and rarely rhabdomyolysis. Acute lesions are transient, reversible and respond favourably to antimalarial drugs [63, 65, 68, 69].

#### 4.3.2. Dengue

Dengue is an arbovirosis transmitted by the infesting bite of the Aedes aegypti mosquito, also known as the “tiger” mosquito. Although the incidence of this disease has increased dramatically worldwide in recent decades, it is still a neglected tropical disease [70]. Dengue is benign in 40–75% of cases. It can affect various organs, including the kidney. Kidney damage is varied and may involve one or more of its structures. Among the renal complications, AKI is the most frequent [70, 71]. In a cohort of 316 Burkinabe with dengue fever, Coulibaly et al. reported 21.2% of AKI [72]. Glomerular biopsies showabnormalities such as hypertrophy and hyperplasia of mesangial and endothelial cells in some glomerular capillaries and focal thickening of the glomerular basement membrane. In other cases, deposition of the immune complex in the glomerulus is a common histological finding [57, 73].

#### 4.3.3. Human Immunodeficiency Virus (HIV) Infection

HIV infection is an important cause of AKI in Africa because of the coexistence of opportunistic infections or co-morbidities such as tuberculosis or because of the toxicity of antiretroviral drugs [6]. Patients with HIV may present with AKI of multifactorial origin, caused by acute tubular necrosis, acute tubulointerstitial nephropathy, thrombotic microangiopathy, AKI during immune restoration syndromes, antiretroviral-induced nephrotoxicity, lymphoplasmacytic interstitial nephritis, nephropathies related to coinfections, and neoplasia [47, 74]. AKI occurs most often in advanced disease and may also be secondary to the use of nephrotoxic drugs common in these patients or to shock. A prospective study of 317 HIV-infected patients in Abidjan (in Ivory Coast) found AKI in 76 patients (24%) [75]. In a cohort of 101 HIV-infected South Africans with AKI, the most common cause of AKI was sepsis (60%), followed by volume depletion and haemodynamic instability [76].

#### 4.3.4. Filariasis

About 859 million people in 50 countries are at risk of lymphatic filariasis. DRC, Madagascar, and Nigeria are among the highly endemic countries in SSA. Lymphaticfilariasis results from infestation by parasitic nematodes (worms) belonging to the family Filaridae. The species Onchocerca volvulus, Wuchereria bancrofti, Loa loa predominate in tropical areas. Loa loa is prevalent in West and Central Africa as well as in the DRC. The incidence of kidney damage during filariasis is not known [47]. The renal damage associated with filariasis is secondary to the deposition of circulating immune complexes in the glomeruli or to their formation in situ [77].

#### 4.3.5. Schistosomiasis

Schistosomiasis affects around 240 million people worldwide [78]. The disease is prevalent in poor areas of the tropics and subtropics [79]. It is caused by the species *Schistosoma intercalatum*, *S. mansoni*, *S. haematobium*, *S. japonicum*, and *S. mekongi*. In SSA, the mansoni, haematobium and intercalatum species are endemic [47]. AKI associated with schistosomiasis is not frequently described in the literature. Chronic glomerular disease is the most frequently reported renal disease [48, 80]. Indeed, AKI is not well studied in schistosomiasis and its pathophysiology is not fully understood. Some authors suggest that it is a nephropathy secondary to deposition of circulating immune complexes or their in-situ formation initiated by schistosomal antigen, while other factors such as autoimmunity may be involved in the progression of the disease [81, 82].

#### 4.3.6. Human African Trypanosomiasis (HAT)

HAT, also known as sleeping sickness, is a vector-borne parasitic disease. The parasite is a protozoan of the genus Trypanosoma that is transmitted to humans by the bite of a tsetse fly, of the genus Glossina. HAT is caused by *Trypanosoma brucei rhodesiense* (East Africa) and *Trypanosoma brucei gambiense* (West and Central Africa), which causes a chronic form of sleeping sickness [83]. Over the past 10 years, more than 70% of reported cases have been in the DRC. One study estimated that in the DRC in 2007, there were 18,592 people with HAT [84]. Glomerular involvement in HAT is secondary to the deposition of circulating immune complexes in the context of chronic antigenicity, including cryoglobulinemia [85, 86].

#### 4.3.7. Leptospirosis

Leptospirosis is a bacterial zoonosis of global importance caused by host-dependent spirochetes of the genus Leptospira (order Spirochaetales). Humans are usually infected through contact with the urine of an infected host, contaminated drinking water or soil, or infected animal tissue [87]. Worldwide, it is estimated that about one million symptomatic cases of human leptospirosis occur each year, resulting in about 60,000 deaths [88]. It is common in tropical areas where humans and animals live in close contact, but the disease has been largely neglected in Africa. In SSA, leptospirosis was the cause of illness in 2.3% to 19.8% of hospitalised patients with fever. Where population-level data were available, leptospirosis was estimated to affect 3–102 persons per 100,000 each year [89, 90]. This zoonosis remains a major public health problem in many SSA countries, particularly because of increasing urbanisation (slums), population growth, climate change and increased risk of flooding leading to an increased burden of leptospirosis in SSA [91]. The kidney is one of the main targets of Leptospira, and kidney damage can occur in 20–85% of patients. Clinical manifestations range from simple urine sediment abnormalities to acute renal failure [57]. Renal tubular and glomerular damage can occur by several mechanisms, such as direct nephrotoxic action of Leptospira, haemodynamic alterations and decreased glomerular filtration rate and rhabdomyolysis [92]. Loss of urinary electrolytes may lead to hypomagnesaemia and hypokalaemia and tubular changes usually precede the decrease in glomerular filtration rate. AKI in leptospirosis is often non-oliguric and hypokalaemic [57, 93]. The aetiopathology of AKI is complex and multifactorial, including the direct effect of the bacteria on renal tissue, hypovolaemia, hypotension, rhabdomyolysis, hyperbilirubinaemia and damage to the endothelial glycocalyx [94].

#### 4.3.8. Ebola Haemorrhagic Fever Disease (EHF)

The extent of AKI during EHF is unknown in SSA. AKI is common in viral diseases and follows the inability of the kidneys to eliminate metabolic waste products and maintain normal fluid homeostasis. In EHF, renal hypoperfusion is the basis of functional AKI. This constellation allows any AKI in the course of EHF to be considered functional in origin. In a cohort of 60 patients with EHF hospitalised at the Macenta treatment centre in Guinea Forestière, 16 (26.7%) of them presented with AKI [95]. AKI in EHF is thought to be a consequence of rhabdomyolysis and is a major concern in EHF [96]. After histopathological examination of tissues from Ebola-infected macaques, Johnson et al. found that AKI during EHF is favoured by several factors: hypovolaemia due to gastrointestinal fluid loss, inflammatory response, and viral injury leading to interstitial nephritis [97].

#### 4.3.9. Coronavirus Disease 2019 (COVID-19)

In December 2019, COVID-19 due to infection with the new SARSCoV-2 (severe acute respiratory syndrome coronavirus (2) virus began to appear. The kidney is one of the targets of the coronavirus. Several studies have reported extremely frequent kidney damage in patients with COVID-19. This damage can present as AKI in over 35% of cases [98, 99]. In Mali, Sy et al. noted that the circumstances in which AKI is discovered are oliguria and total macroscopic haematuria [100]. The mechanisms and type of renal damage during infection by the new coronavirus remain to be determined [100]. Polymerase Chain Reaction analysis of organs from deceased COVID-19 patients revealed that the kidneys are among the most frequently affected target organs of the virus, after the lungs [101]. The high frequency of renal involvement is explained by the presence of the viral receptor ACE2 (angiotensinconverting enzyme (2) and its co-receptors on the surface of renal cells. The virus is thus likely to affect several compartments of the kidney, including the glomeruli, the endothelia and the proximal tubule; some data suggest APO L1 genotype may be associated with worse AKI outcomes [102, 103]. A South African study reported 33.9% AKI in 1102 patients with COVID-19 hospitalised in two tertiary hospitals in South Africa [104].

#### 4.3.10. Chikungunya Virus Infection

Chikungunya is a viral disease transmitted to humans by infected mosquitoes. It is caused by the chikungunya virus (CHIK-V). CHIK-V is an RNA virus belonging to the *Togaviridae* family and the alphavirus group, which comprises 28 viruses including *Chikungunya*, *O'Nyong Nyong*, *Ross River*, *Sindbis* and *Mayaro*. There is an African strain of CHIK-V with two variants (East, Central and Southern Africa; West Africa) and an Asian strain [105]. In Reunion Island, the study by Economopoulou et al. reported 121 (20%) cases of AKI in a series of 610 adults infected with CHIK-V [106]. In 2019, Missamou et al. reported 4 first cases of AKI during CHIK-V infection in Republic of Congo. These authors point out that all patients presented with rhabdomyolysis with a significant elevation of lactate dehydrogenase compared to creatinine phosphokinase [107]. The renal histopathological findings were mainly acute interstitial nephritis, glomerular congestion/edema and acute tubular necrosis [108].

#### 4.3.11. Other Infections

Several other bacterial, viral, and parasitic infections have been found in the literature to cause AKI in adults in SSA. These include typhoid and paratyphoid fevers [109–111], leishmaniasis [112, 113], and Rift Valley fever [114, 115].

### 4.4. Nephrotoxic Agents

Nephrotoxic AKI is much more common in SSA and is mainly due to intoxication by plants, chemicals, and snake envenomation. AKI associated with these nephrotoxic agents is an important clinical problem that mainly affects healthy, economically active young adults [116]. Traditional herbal medicines are commonly found in SSA and are important causes of AKI [6, 48]. The ingestion of many toxic plants, namely *Callilepis laureola*, *Aloe capensis*, poisonous mushrooms, *Euphorbia matabalensis* and other medicinal plants, also contributes to the high incidence of nephrotoxic AKI in SSA [5, 6, 117–121]. Apart from secondary plant intoxications, we also find envenomations by snakebite [111]. A Togolese study of 376 patients hospitalised for snakebite found that 12.2% had AKI, all classified as KDIGO stage 3 [122]. The suspected mechanisms were rhabdomyolysis, thrombotic microangiopathy and disseminated intravascular coagulation in 50%, 33%, and 17% of cases respectively and the case fatality of AKI during snakebite envenomations was 58.7% [122]. Other nephrotoxic agents found in the African literature are chemicals such as naphthalene [123], and paraphenylene-diamine [124]. The use of skin lightening (bleaching) creams containing Mercury has also been reported to cause kidney damage in many parts of SSA [125–127].

### 4.5. Obstetrical Complications

Although there is no consensus on the exact definition of obstetric AKI [128]; it should be noted that it encompasses all causes of acute renal function impairment from early pregnancy to three months postpartum. It is one of the serious complications, putting at risk the vital prognosis of the mother and the fetus. In developing countries, obstetric AKI is still common with an incidence of 4.2–15% [129]. Cooke et al. in a cohort of 322 women admitted to the obstetric wards of a tertiary hospital in Blantyre, Malawi, reported 8.1% AKI and found that the main causes of AKI were preeclampsia/eclampsia (73.1%), antepartum haemorrhage (11.5%), and sepsis (11.5%) [130]. At the National Hospital of Nouakchott (Mauritania), Abdelkader et al. recorded a prevalence of 6.09% of postpartum AKI and the aetiologies were dominated by preeclampsia (61.76%) followed by obstetric haemorrhage (38.23%) [131].

### 4.6. Obstructive Uropathy

In SSA, although not widely reported, obstructive uropathy has been found in somestudies to be an important cause of AKI [39, 132]. According to the literature, between 6% and 20% of AKI cases had obstructive uropathy [39, 48, 111]. The main causes were pelvic cancer and kidney stones, prostate cancer in men, and cervical, endometrial and ovarian cancer in women [39, 60, 111, 133, 134].

### 4.7. Perioperative AKI

The perioperative period is a renal risk situation because anaesthesia and surgery are themselves sources of renal stress. The hormonal and haemodynamic changes associated with anaesthesia, together with surgical stress and mechanical ventilation, lead to deregulation of the renal system, progressing to a profile of functional renal failure. Perioperative AKI is often multifactorial in origin, combining iatrogenic, haemodynamic and surgical factors. Its prevention is based on haemodynamic optimisation (venous return, cardiac output, vascular resistance), avoidance of nephrotoxic drugs, but also on knowledge of surgical times to anticipate a possible deterioration in renal haemodynamics [135]. Postoperative AKI is the second most common cause of hospital-acquired AKI, after septic shock and before cardiogenic shock [136]. In Nigeria, in a single-centre prospective cohort of 219 patients undergoing major surgery, the incidences of AKI were 18.7% at 24 hours and 17.4% on postoperative day 7, while the cumulative incidence of AKI was 22.5% at 1 week postoperatively. AKI was associated with a mortality of 20.4% [137]. In a cohort of 223 trauma patients hospitalised at the tertiary trauma referral centre in the central region of Malawi, 14.4% had developed AKI within the first 3 days of hospitalisation [138].

## 5. Management and Prognosis of AKI in Sub-Saharan Africa

AKI is a frequent and formidable complication because it is costly to manage and associated with a high rate of prolonged hospitalisation and in-hospital mortality [139]. The development of AKI in hospitalised patients increases direct and indirect healthcare costs. AKI is associated with increased investigation and monitoring, unplanned or longer ICU stays, prolonged hospital stays and increased risk of early rehospitalisation [7]. While definitions of AKI and its classifications vary, the diagnostic approach is always the same, based on clinical, radiological and biological findings with creatinine and diuresis as the usual markers, despite the criticism [36, 37, 140].

The aetiologies are extremely varied and their management is, in most cases, a medical and/or surgical emergency. In addition to symptomatic and etiological measures, supportive therapy (haemodialysis, peritoneal dialysis) in the acute phase has been shown to improve the prognosis of AKI patients. The choice of dialysis is above all a decision based on its availability and the expertise of the team, the knowledge of its advantages and limits, allows us to adapt more precisely to the indications of extrarenal purification and to its requirements [16, 17]. As in the rest of the world, the need for renal health care and renal replacement therapy is increasing in SSA. Access to these treatments remains limited in SSA due to a real lack of dialysis facilities [141].

Haemodialysis is the treatment for kidney disease available in SSA countries, but accessibility is limited by financial means. When the cost of haemodialysis can equal the annual income of an African family, difficult decisions have to be made; in most cases, only a few haemodialysis sessions can be provided and the family is not able to commit to paying for long-term treatment [142]. Peritoneal dialysis is often the only life-saving and more affordable alternative to haemodialysis [143]. Despite the many technical innovations in renal replacement therapy, mortality remains high due to the ageing of the population and visceral failure, especially in a septic context [139]. AKI remains associated with complications such as high in-hospital mortality and a high risk of developing CKD in long-term survivors. The overall hospital mortality of AKI varies between studies and countries. There are several possible explanations for the variation in AKI mortality rates: difference in patient admission sites (intensive care unit, general medical ward or surgical ward), study design and the underlying disease of the patients (some authors exclude patients with a history of CKD) [139]. Mortality rates of 36.9%, 44.4%, and 58% were reported in Cameroon [34], Malawi [33], and the DRC [35], respectively.

## 6. Prevention of AKI in Sub-Saharan Africa

In the absence of effective therapeutic interventions on established AKI and due to morbidity and mortality associated with AKI, we can only rely on AKI prevention and early diagnosis to reduce its incidence and detrimental consequences. Sustainable Development Goal 3 commits to ending the epidemics of AIDS, tuberculosis, malaria and neglected tropical diseases and combat hepatitis, waterborne diseases and other communicable diseases by 2030 [144]. In SSA, prevention of AKI and its complications is possible and can be achieved through interventions at individual, national and regional levels. It can be achieved through simple and inexpensive means, including identification of patients at risk, avoidance of nephrotoxic drugs and correction of patients' blood volume status. However, this fundamental action can only be achieved through public awareness and serious involvement of the medical community and public authorities [17].

The gold-standard of AKI prevention in SSA countries remains the overall improvement in hygiene conditions, in particular greater access to drinking water and programs for vaccination and eradication of common infectious agents (for example through the use of mosquito nets and antimalarial drugs). Many of AKI episodes begin in the community setting, so health care professionals should identify at-risk patients and implement preventive interventions to decrease the incidence of AKI. At hospital admission, patients should also be screened for AKI risk. In high-risk patients, the early correction of modifiable risk factors should be considered in order to prevent AKI occurrence. Encouraging better detection of patients at risk must be done by primary care physicians. This therefore requires regular measurements of renal function and proteinuria to be carried out in elderly, hypertensive or diabetic patients, and especially when introducing or changing the dosage of a potentially nephrotoxic medicinal product whose dose administered must be adapted to the level of renal function of the patient.

## 7. Conclusion

AKI is a public health problem in SSA because of the burden of disease (especially AKI related to viral, bacterial, and parasitic infections, diarrhoeal diseases, and nephrotoxic agents), the late referral of patients to health care facilities, and the lack of resources (human, material) to manage AKI patients established in many countries. The profile of AKI patients in resource-limited countries is very different from that of more industrialised countries. In addition, there is a need to prevent AKI and provide renal support for patients requiring dialysis management. AKI is a health problem of high proportions and probably deserves the same attention as other common medical problems. AKI is a growing problem, but its true incidence is unknown. From a global perspective, there is a clear need to understand more precisely the epidemiology of AKI. The use of standardised definitions and descriptions of existing at-risk and high-risk populations, both in community and institutional settings in developing and underdeveloped countries, is the first step to improving outcomes.

## Figures and Tables

**Table 1:** KDIGO 2012 staging criteria for acute kidney injury [19].

Stage	Serum creatinine	Amount of urine produced

1	1.5–1.9 times the initial value; OR ≥ 0.3 mg/dL (26.52 micromoles/L)	<0.5 mL/kg/h for 6–12 h
2	2–2.9 times the initial value	<0.5 mL/kg/hour for ≥12 h
3	≥3 times the initial value; OR ≥ 4.0 mg/dL (353.60 micromoles/L); OR if renal replacement therapy is initiated; OR in patients <18 years, decrease in eGFR to <35 ml/min per 1.73 m^2^	<0.3 mL/kg/h for ≥24 h OR anuria for ≥12 h

## Data Availability

The data used to support the findings of this study are available from the corresponding author upon request.

## Abbreviations

95% CI: 95% confidence intervals
ADQI: Acute dialysis quality initiative
AKI: Acute kidney injury
AKIN: Acute kidney injury network
AOR: Adjusted odds ratio
CHIK-V: Chikungunya virus
CKD: Chronic kidney disease
COVID-19: Coronavirus 2019
DRC: Democratic republic of the congo
eGFR: Estimated glomerular filtration rate
EHF: Ebola haemorrhagic fever disease
HAT: Human african trypanosomiasis
HIV: Human immunodeficiency virus
KDIGO: Kidney disease: improving global outcomes
RIFLE: Risk, injury, failure, loss of kidney function, and end-stage kidney disease
SCD: Sickle cell disease
SSA: Sub-Saharan Africa.
